# Supplementary material for: Causal Relationship between Aging and Anorexia Nervosa: A White-Matter-Microstructure-Mediated Mendelian Randomization Analysis
Source: Biomedicines. 2024 Aug 16;12(8):1874. doi: 10.3390/biomedicines12081874 (PMC11351342; doi:10.3390/biomedicines12081874)
Supplement: Supplementary file 1 [file biomedicines-12-01874-s001.zip › Supplementary Figure/Figure S8/Leaveoneout plot of MR analysis for TL to ubm-b-1478.pdf]

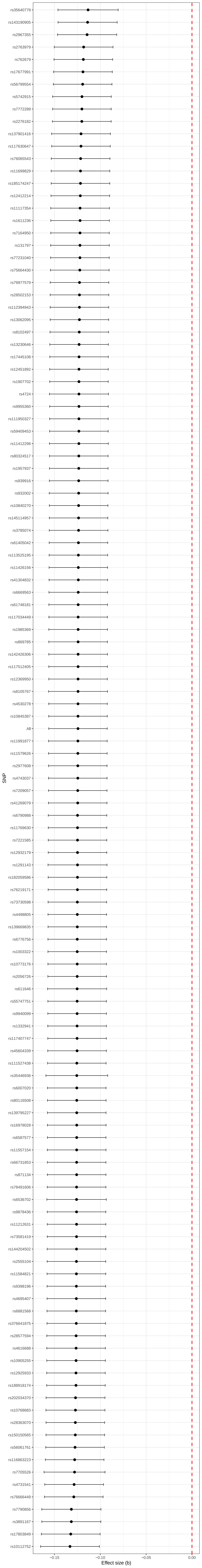

rs35640778
rs143190905
rs2967355
rs2763979
rs762679
rs17677991
rs56799554
rs5742915
rs7772289
rs2276182
rs137901416
rs117630647
rs76065543
rs11699829
rs185174247
rs12412214
rs11117354
rs1611236
rs7164950
rs131797
rs77231040
rs75664430
rs79977579
rs28502153
rs112394943
rs13062095
rs8102497
rs13230646
rs17445108
rs12451892
rs1907702
rs4724
rs9955360
rs111950327
rs59409453
rs11412296
rs80324517
rs1957937
rs939916
rs932002
rs10840270
rs145114957
rs3785074
rs61405042
rs113525195
rs11426156
rs41304832
rs6669563
rs61748181
rs117034449
rs1985369
rs869785
rs142426306
rs117512405
rs12369950
rs8105767
rs4530278
rs10845387
All
rs11991877
rs11579626
rs2977608
rs4743037
rs7209057
rs41269079
rs6790988
rs11769630
rs7221585
rs12932179
rs1291143
rs182059586
rs76219171
rs73730598
rs4498805
rs139669835
rs6776756
rs1003322
rs10773176
rs2056726
rs611646
rs55747751
rs9940099
rs1332941
rs117407747
rs45604339
rs111527438
rs35446936
rs6007020
rs80116508
rs139795227
rs16978028
rs6587577
rs11557154
rs66731853
rs871134
rs78491606
rs6536702
rs9878436
rs11212631
rs73581419
rs144204502
rs2555104
rs11584821
rs9398196
rs4695407
rs6881568
rs376641875
rs28577594
rs4616688
rs10905255
rs12925933
rs188918174
rs202034370
rs10768683
rs28363070
rs150150565
rs56061761
rs116863223
rs7705526
rs4731541
rs76666449
rs7790856
rs3891167
rs17803849
rs10112752
SNP
-0.15
-0.10
-0.05
0.00
Effect size (b)
